# Pea Seed Priming with Pluronic P85-Grafted Single-Walled Carbon Nanotubes Affects Photosynthetic Gas Exchange but Not Photosynthetic Light Reactions

**DOI:** 10.3390/ijms25147901

**Published:** 2024-07-19

**Authors:** Sashka Krumova, Svetozar Stoichev, Daniel Ilkov, Velichka Strijkova, Vesela Katrova, Ana Crespo, José Álvarez, Elvira Martínez, Sagrario Martínez-Ramírez, Tsonko Tsonev, Petar Petrov, Violeta Velikova

**Affiliations:** 1 Institute of Biophysics and Biomedical Engineering, Bulgarian Academy of Sciences, 1113 Sofia, Bulgaria; sashka.b.krumova@gmail.com (S.K.); thylakoid85@gmail.com (S.S.); 2 Institute of Plant Physiology and Genetics, Bulgarian Academy of Sciences, 1113 Sofia, Bulgaria; d_ilkov@bio21.bas.bg (D.I.); tsonev@gmail.com (T.T.); 3 Institute of Optical Materials and Technologies, Bulgarian Academy of Sciences, 1113 Sofia, Bulgaria; vily_strij@abv.bg (V.S.); vlozanova@iomt.bas.bg (V.K.); 4 Instituto de Estructura de la Materia (IEM—CSIC), 28006 Madrid, Spain; a.crespo.i@csic.es (A.C.); sagrario@iem.cfmac.csic.es (S.M.-R.); 5 Ingeniería Agroforestal, ETSIAAB, Universidad Politécnica de Madrid, 28040 Madrid, Spain; jose.alvarez.sanchez@upm.es (J.Á.); elvira.martinez@upm.es (E.M.); 6 Institute of Polymers, Bulgarian Academy of Sciences, 1113 Sofia, Bulgaria; ppetrov@polymer.bas.bg

**Keywords:** leaf pigments, photosynthetic gas exchange, nanomaterials, photosynthesis, seed germination, stomatal conductance

## Abstract

Nanotechnology is rapidly advancing towards the development of applications for sustainable plant growth and photosynthesis optimization. The nanomaterial/plant interaction has been intensively investigated; however, there is still a gap in knowledge regarding their effect on crop seed development and photosynthetic performance. In the present work, we apply a priming procedure with 10 and 50 mg/L Pluronic-P85-grafted single-walled carbon nanotubes (P85-SWCNT) on garden pea seeds and examine the germination, development, and photosynthetic activity of young seedlings grown on soil substrate. The applied treatments result in a distorted topology of the seed surface and suppressed (by 10–19%) shoot emergence. No priming-induced alterations in the structural and functional features of the photosynthetic apparatus in 14-day-old plants are found. However, photosynthetic gas exchange measurements reveal reduced stomatal conductance (by up to 15%) and increased intrinsic water use efficiency (by 12–15%), as compared to hydro-primed variants, suggesting the better ability of plants to cope with drought stress—an assumption that needs further verification. Our study prompts further research on the stomatal behavior and dark reactions of photosynthesis in order to gain new insights into the effect of carbon nanotubes on plant performance.

## 1. Introduction

Interest in carbon nanotube application in the fields of plant biotechnology, bioengineering, and agronomy is prompted by the fact that they can be suitable nanomaterials for plant cell transfection and for the targeted delivery of a variety of molecules (either loaded in the nanotube core or attached to the nanotube surface by chemical functionalization) to different plant tissues. The transfer of only a miniscule amount of these compounds to intact plant compartments would have a huge ecological impact due to the utilization of small amounts of materials, significant reduction in environmental pollution, and economic benefits. The internalization of genetic material, nutrients, biostimulants, antioxidants, and other beneficial substances for plant metabolism within plant tissues via nanocarriers is also expected to have a large impact on stress tolerance and survival in adverse climate conditions, and thus on crop yield (these applications are thoroughly reviewed in [1]. In addition to serving as nanovehicles, bare carbon nanotubes have also been shown to act as plant growth regulators, since their interaction with seeds, roots, and leaves affects multiple metabolic processes, gene expression, protein synthesis, hormonal levels, pigments, and photosynthetic function within plants [2,3,4,5,6,7,8].

Nanotubes are also known to be able to directly interact with chloroplasts, photosynthetic pigments, and complexes [9,10,11,12,13]. Giraldo et al. [9] showed that single-walled carbon nanotubes (SWCNT) diffuse within the chloroplast membrane and donate electrons to the photosynthetic electron transport chain. Enhanced photosynthesis was also reported upon the injection of chloroplasts with SWCNT [9]. Isolated reaction centers and light-harvesting complexes have been reported to form hybrid structures with carbon nanotubes [10,11,12,13]. These in vitro studies, however, do not answer the question as to whether nanotubes have the capacity to exert long-lasting effects on plant growth, photosynthetic performance, and the yield of intact plants. Therefore, a variety of suitable experimental approaches based on leaf and seed treatments have been developed and are feasible to apply in real-life conditions.

There are many data in the literature describing the effects of leaf exposure to different nanomaterials, including SWCNT [14,15,16]. Specifically, with regard to P85-SWCNT, we have recently shown that doses below 100 mg/L appear safe for the plant, while the concentration of 300 mg/L impairs the structural and functional characteristics of the photosynthetic machinery [17,18,19]. However, the available data regarding the effect of SWCNT on seeds of different plant species [2,4,5,6,7,8,20] do not allow for the drawing of definite conclusions regarding their integral mode of action in plants. A suitable tool for the characterization of treated seed germination and root growth is hydroponic culturing, which is also a valuable tool in studies related to the development of soilless farming approaches [21]. However, understanding the nanomaterial/seed and plant interactions in conditions close to the natural ones requires soil substrate growth or the treatment of already-developed plants. Therefore, the use of different treatment and cultivation approaches is essential for an accurate understanding of plant responses.

Similar to their multi-walled analogues, SWCNT can be loaded or functionalized with different molecules but have smaller sizes, which is expected to enhance their penetration through different barriers in the plant (the seed coat, plant cell walls, and membranes). This might have an advantageous effect due to the enhanced transportation through the plant’s vascular system, which allows localization in various plant compartments where they can exert their specific function. Alternatively, it can have a deleterious effect due to the nanotube-induced mechanical damage (piercing) of the cell walls and membranes. Seed exposure to bare SWCNT (up to 40 mg/L) dispersed in water has been reported to have stimulating effects on seed germination [2,4,6,7,8], seminal root growth [5], and the biomass of aerial parts [2,4,7] in various plant species, while higher doses have generally exhibited negative/toxic effects. SWCNT have been shown to be able to penetrate the seed coat and roots [2,5] and to be active players in reactive oxygen species (ROS) metabolism and the expression of genes related to antioxidant defense, root and chloroplast development, and in the biosynthesis of proteins, phenolics, proline, and plant hormones [5,6,7,8]. Recently, we have utilized seed priming [20] and leaf spraying [17] procedures with P85-SWCNT to explore their potential for plant growth modulation. Our previous study showed that, in hydroponic systems, seed priming with concentrations below 100 mg/L of P85-SWCNT did not impair seed germination, early growth stages of pea plants, or their photosynthetic activity, while higher concentrations exerted pronounced negative effects [20].

The aim of the present study is to evaluate the effect of P85-SWCNT seed priming on plant growth in conditions that mimic natural ones. To achieve this goal, for the first time, we explored the potential of P85-SWCNT to affect early plant growth and functional status, particularly photosynthetic performance, in soil gardening conditions. Another novelty of our study is the evaluation of the possible correlation between seed physical characteristics (studied by means of atomic force microscopy (AFM) and Raman spectroscopy) and the plant photosynthetic activity of developed plants. The present study provides further valuable information on the possible effects of nanomaterials on plants grown in soil substrates and a better understanding of the mode by which the tested priming protocols would moderate seed germination and seedling development in nature.

## 2. Results and Discussion

### 2.1. Seed Germination of Soil Gardening Pea Plants

We followed the seed germination process of variants primed either with H_2_O or with 10 and 50 mg/L P85-SWCNT on soil substrate for a period of 5 days. The germination process was retarded for the two P85-SWCNT primed variants, due to a larger mean germination time (*MT*) and lower mean germination rate (*MR*), resulting in lower (by 10–19% on the average) germination (*G*) values. The seed synchronization (*Z*) was also negatively affected by the treatments with the applied nanomaterials (Table 1).

These inhibitory effects on seed germination were not observed for hydroponic growth [20], most probably due to the distinct environment (soil/water) and nutrition supply in these two different environments that seeds face during germination.

### 2.2. Leaf Pigments and Photosynthetic Performance

The functional characteristics of developed leaves were evaluated, with emphasis on photosynthetic operation, in order to gain more insight into the development of soil gardening plants after P85-SWCNT seed priming. The total leaf chlorophyll (Chl) and flavonoid abundance were not affected by the applied seed priming procedures. The nitrogen balance index (NBI) was only slightly reduced (less than 5%) by the P85-SWCNT treatments compared to the hydro-priming (Table 2).

The net photosynthetic rate (*A_N_*) after the P85-SWCNT priming procedure was affected only marginally, within 7% of the value recorded for the hydro-primed variant (Table 3). In our previous study, we showed that no substantial differences in *A_N_* were observed between the H_2_O- and 10 mg/L P85-SWCNT-sprayed pea plants, while higher concentrations of P85-SWCNT (above 100 mg/L) significantly reduced *A_N_* [17]. This demonstrated that P85-SWCNT can interact with plant systems in various ways (e.g., photosynthetic proteins, pigments, chloroplasts), potentially enhancing or inhibiting the photosynthetic process [9,22,23], and that this strongly depends on the mode of nanomaterial application.

Here, we observed an apparent decrease (by up to 15%) in the stomatal conductance (*g_s_*) of plants developed from P85-SWCNT-primed seeds. Contrary to stomatal conductance, the intrinsic water use efficiency (*iWUE*) of P85-SWCNT plants increased by 12–15%, compared to hydro-primed ones (Table 3), suggesting better control over water loss through stomata, thereby helping plants to maintain hydration and continue physiological processes under potential drought conditions [24]. The lower values of *g_s_* in plants developed from P85-SWCNT-primed seeds might be associated with specific alteration in plant hormone levels that regulate the stomata development and patterning (reviewed in [25]). The lower stomatal conductance might also be due to stronger cutinization of the outer elastic edge of stomata, as observed by us after pea leaf spraying with 300 mg/L P85-SWCNT [17]. Leaf stomata play a crucial role in regulating water and CO_2_ exchange, and it is worth investigating further whether the utilized nanoparticles are also able to affect stomatal behavior. Plant survival and adaptation to unfavorable environmental conditions strongly depend on the effective stomatal control, allowing for optimal CO_2_ uptake and water loss balance [26,27,28]. Consequently, alteration in this process might have a crucial effect on plant fitness and survival. It is also worth noting that *iWUE* was higher in P85-SWCNT variants compared to the hydro-primed ones. *iWUE* is an important eco-physiological trait that provides a reliable assessment of how effectively water is saved by plants coping with drought stress [24]. Therefore, our data might indicate that P85-SWCNT seed priming could provoke the development of a more efficient water saving strategy and higher capacity of photosynthesis of P85-SWCNT plants under stressful conditions. However, this assumption needs further verification.

Chl fluorescence parameters recorded using the PAM imaging technique on intact leaves showed no significant changes in the functionality of the photosynthetic apparatus upon growth of primed seeds on soil substrate (Table 4), similar to hydroponic growth conditions, where those parameters varied by less than 10% of the value recorded for the hydro-primed variant [20].

The distribution of Chl *a* and *b* within grana and stroma lamellae and the extent of grana stacking were not affected by the P85-SWCNT priming protocols (Table 5), revealing that the applied seed treatments did not alter the structural organization of the photosynthetic apparatus. In addition, the different priming procedures did not change the level of malondialdehyde (MDA) in the thylakoid membranes (Table 5), strongly suggesting that there is no modification with respect to the extent of oxidation of fatty acids and/or the fatty acid composition of thylakoid membrane lipids [29] induced by P85-SWCNT priming procedures. This also excludes extensive ROS accumulation due to P85-SWCNT seed treatment, since it would inevitably exert a strong effect on the photosynthetic machinery and the MDA level in thylakoids.

### 2.3. Seeds Topology Studied by AFM

Next, we investigated whether the effects observed on the seed germination and the functional activity of pea plants are associated with altered physical seed properties.

**Figure 1:**
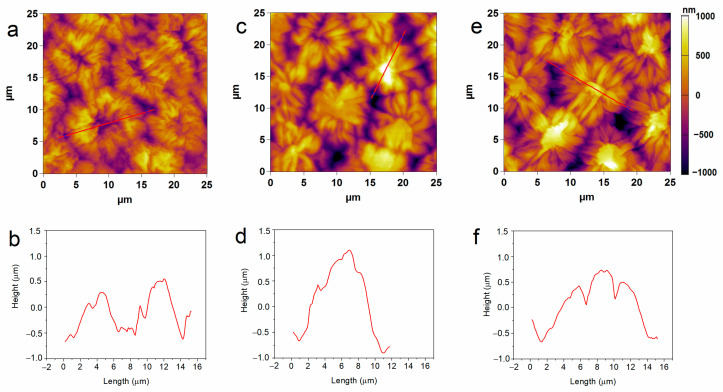
Typical 2D AFM images of hydro- (**a**), 10 mg/L P85-SWCNT- (**c**), and 50 mg/L P85-SWCNT (**e**)-primed seed surface and respective height profiles of the structures marked with red lines (**b**,**d**,**f**).

The structural characteristics of pea seeds upon hydro-priming and P85-SWCNT priming were first assessed by atomic force microscopy (AFM). Nanoimaging in air resolved typical testa texture in hydro-primed samples, i.e., symmetric circular formations with sizes of about 10 microns, defined by macrosclereid arrangement [30,31,32]. However, the 10 and 50 mg/L P85-SWCNT-primed seeds exhibited distorted structures with an altered symmetry and often with a high central component (>800 nm, Figure 1). The estimated average root mean square (RMS) roughness values for an area of 600–800 μm^2^ were higher for the 10 mg/L (322 ± 15 nm) and 50 mg/L (295 ± 18 nm) P85-SWCNT treatments than for the hydro-primed variant (259 ± 20 nm), but the differences were not statistically significant. In the obtained AFM images, individual nanotubes were not resolved, most likely due to technical limitations, namely the cantilever tip radius of 7 nm, which did not allow for the visualization of thin objects such as P85-SWCNT, with a diameter of about 1 nm [17].

### 2.4. P85-SWCNT–Seed Interaction Probed by Raman Spectroscopy

**Figure 2:**
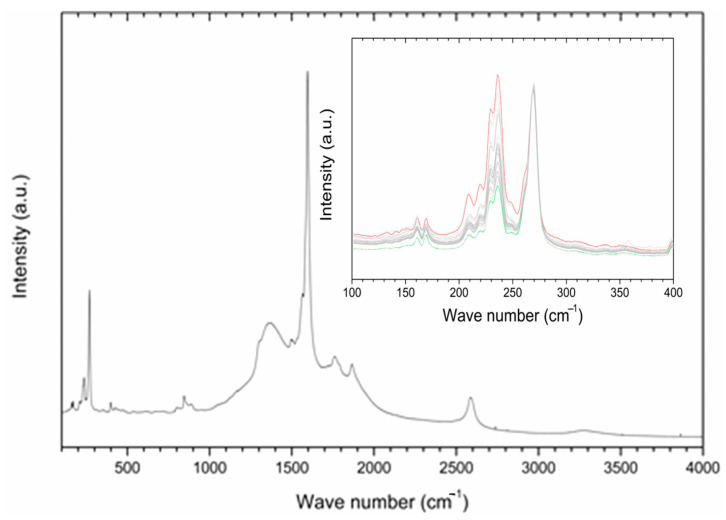
A typical Raman spectrum of P85-SWCNT dispersion with a concentration of 10 mg/L; inset—zoomed region in the range 100−400 cm^−1^ for various analyzed spots on the seed coat surface. For clarity, the spectra in the inset are normalized to the peak at 268 cm^−1^, and the highest and lowest signals at 236 cm^−1^ are shown in red and green, respectively.

Raman spectroscopy was employed in order to gain information about the interaction of P85-SWCNT with seeds and their distribution within the different seed compartments. First, P85-SWCNT dispersions used for priming were characterized as reference. The acquired spectra were well defined and were successfully assigned to P85-SWCNT (Figure 2) (according to [33]). No other signals in addition to P85-SWCNT were identified. In order to study the reproducibility of the spectra, 30 different points of the P85-SWCNT were acquired. Raman signals remained quite constant in their wavenumber as well as in their intensity. However, the lower region of the spectra presented variability regarding the relative intensity of the signals at 235 and 268 cm^−1^ (Figure 2).

**Figure 3:**
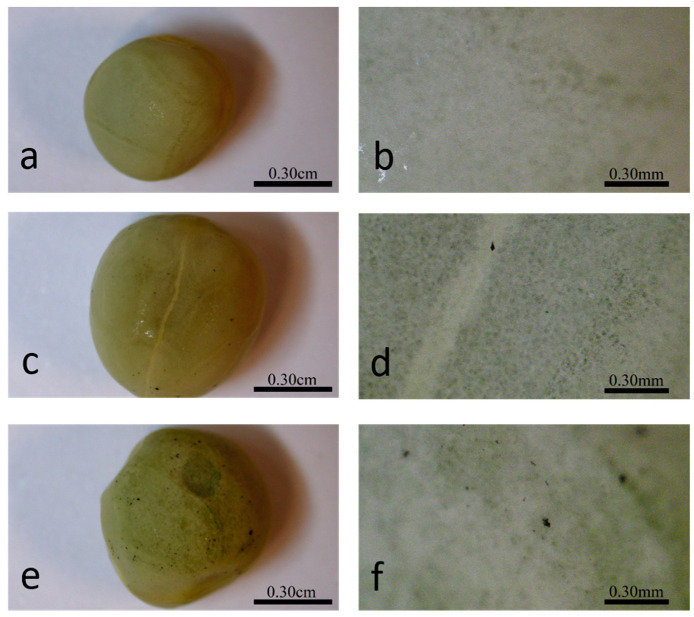
Different magnifications of the seed coat of hydro- (**a**,**b**), 10 mg/L P85-SWCNT- (**c**,**d**), and 50 mg/L P85-SWCNT (**e**,**f**)-primed seeds.

**Figure 4:**
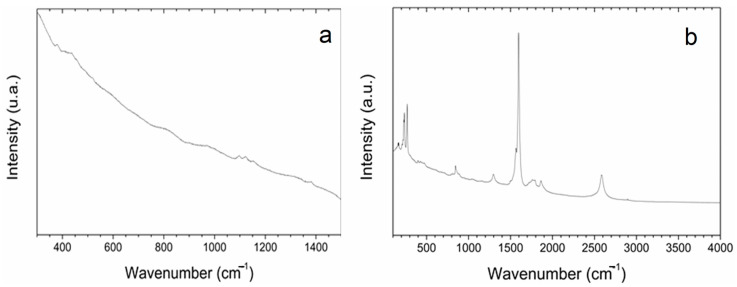
Raman spectra of pea seed coats recorded for hydro- (**a**) and 10 g/L P85-SWCNT (**b**)-primed variants with focus on surface-located P85-SWCNT.

Optical imaging of the primed seeds revealed P85-SWCNT aggregates on the seed coat with different sizes (Figure 3), and the largest ones were even visible with the naked eye. Raman spectra of the seed coat without treatment were difficult to analyze due to the high fluorescence signal of the seed coat. Small bands at 380, 1097, 1123, and 1381 cm^−1^ were identified (Figure 4a), although no assignment was possible. However, when Raman spectroscopy was performed specifically on the spots with P85-SWCNT aggregates, the acquired spectra had very good resolution, i.e., only signals from P85-SWCNT were detected, without contribution from other signals from the seed surface (Figure 4b).

**Figure 5:**
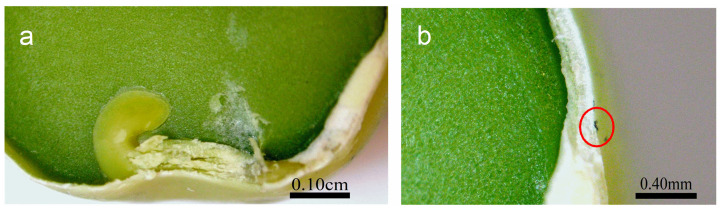
Cross-section of the pea seed primed in 10 mg/L P85-SWCNT dispersion with a focus on the germen and endosperm (**a**) and the seed coat with a P85-SWCNT aggregate on top, as indicated by a red circle (**b**).

**Figure 6:**
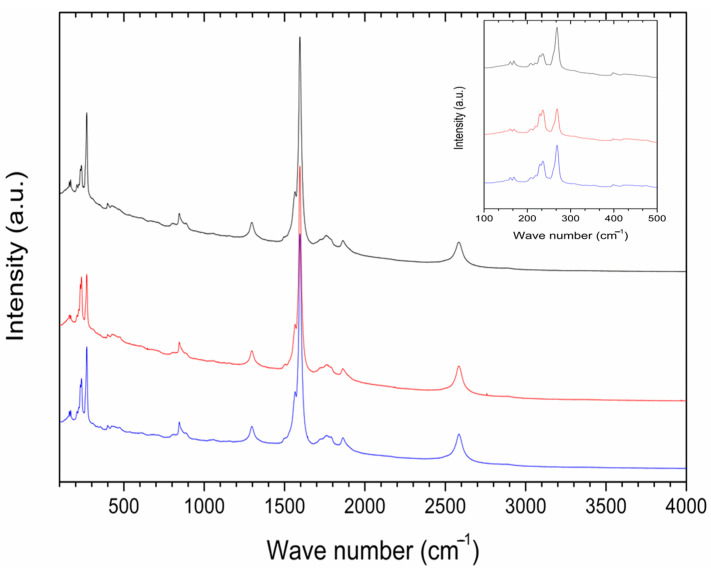
Raman spectra of three distinct spots (plotted in different colors) with P85-SWCNT aggregates analyzed throughout the coat of the seed; inset—magnification of the 100–500 cm^−1^ region of the spectra.

A cross-section in the central part of P85-SWCNT-primed seeds revealed that no internal black spots were found within the resolution limit of the instrument, and that the large nanotube aggregates visible on the seed coat surface were confined to this specific location (Figure 5). It should be noted that Raman spectroscopy is highly sensitive to SWCNT despite the strong fluorescence signal. Thus, no evidence for scattering of P85-SWCNT inside the seed was obtained, as evidenced by the lack of Raman signal in the endosperm of the primed seeds (Figure 6).

The collected micro-Raman spectroscopy data revealed important aspects of the nanotube–seed interaction mode. The Raman spectra of P85-SWCNT presented a variation in the relative intensity of the signals at 235 and 268 cm^−1^. This spectral region of the P85-SWCNT spectra is assigned to the radial breathing mode (RBM) [34]. According to the literature, the intensities of Raman signals in P85-SWCNT are related with the electron–phonon coupling, and the differences in the relative intensity of the RBM region reflect a shift of transition energy [34]. Additionally, the increased intensity of the 268 cm^−1^ band must be associated with P85-SWCNT bundling [35]. As the variation in the relative intensities of the Raman signals was observed in both the P85-SWCNT solution (Figure 2) and the P85-SWCNT spots located on the seed coat (Figure 6), electronic interaction between the P85-SWCNT and the pea was discarded. Chemical interaction between the P85-SWCNT and the pea seed constituents was also discarded, since there was no variation in the spectra when comparing the P85-SWCNT in solution and on the seed cover or seed cross-section. Therefore, only physical interaction between those nanoparticles and seeds could be anticipated, which is most probably the trigger of the observed structural changes in the seed texture of P85-SWCNT-primed seeds, as evidenced by AFM (Figure 1).

The observed structural alteration in seed surface properties apparently did not affect the capability of seeds to absorb water, as evidenced by the unaltered extent of imbibition of hydro- and P85-SWCNT-primed seeds evaluated recently by us [20]. The P85-SWCNT-induced structural remodeling of the seed surface, however, might affect the uptake of larger molecules from the soil substrate and thus modulate the nutrition status of germinating seeds and developing seedlings.

The present work provides valuable insight into the mode of action of SWCNT on seeds, which is scarce in the literature, but also allows for a direct comparison of the capacity of P85-SWCNT to alter pea plant development and functionality via two different experimental approaches—seed priming and leaf spraying. We have demonstrated that P85-SWCNT physically interact with both the leaves and the seed coat: (i) they are able to modify the leaf morphology but also structurally and functionally affect the leaf tissues and chloroplasts [17,18,19]; (ii) they modify the seed coat topology without penetrating deep into the seed interior and do not induce important changes in the photosynthetic reactions of plants derived from primed seeds (this work).

The development of nanomaterial-based strategies for plant growth optimization is at the forefront of agronomy and plant bioengineering research but still has limited practical application. A major obstacle is the lack of knowledge of the basic principles underlying the specific interactions between the large variety of available nanomaterials and the myriads of plants species, as well as the effects they exert on the environment and humans. Therefore, further studies revealing the mode of interaction of nanomaterials with plants are essential in order to gain sufficient fundamental knowledge for the further development of innovative approaches for sustainable agriculture.

## 3. Materials and Methods

### 3.1. Seed Priming and Growth Conditions

For each individual priming experiment, 50 seeds of *Pisum sativum* (var. RAN-1, Florian, Bulgaria) were soaked in 50 mL distilled water (hydro-priming), and 10 mg/L and 50 mg/L P85-SWCNT dispersion (prepared as in [17]) and gently rotated for 6 h. After this period, the seeds were left to dry to their original weight (typically for 12–14 days). Immediately before sowing into a soil substrate (TS3 substrate for vegetables and bedding plants, Klasmann-Deilmann GmbH, Geeste, Germany; growth substrate composition: 86% soil; 12% sand and perlite 2%), seedlings (from each bath of 50 seeds) were grown for a period of 14 days in a climatized chamber at 22 °C, 12/12 h photoperiod, 65% relative air humidity and 400 μmol m^−2^ s^−1^ photon flux density (provided by SMD LED 6500K lamps, Velamp Industies, Milano, Italy). “Pluronic” P85 was obtained from BASF (Ludwigshafen/Rhein, Germany) and SWCNT with >77% carbon and diameter × length 0.7–1.1. nm × 300–2300 nm were purchased from Sigma-Aldridge (Darmstadt, Germany). The priming experiments and the following growing traits were repeated 4 times.

### 3.2. Characterization of Seeds and Early Plant Growth

#### 3.2.1. Seed Germination

The seed germination process was characterized on the basis of shoot emergence for the initial 5 days after sowing. For this purpose, the following parameters described by [36] were utilized: germinability (*G*), mean germination time (*MT*), mean germination rate (*MR*), and synchrony of the germination process (*Z*).

#### 3.2.2. Atomic Force Microscopy on Seeds

AFM imaging was performed using an Atomic Force Microscope (MFP-3D, Asylum Research, Oxford Instruments, Santa Barbara, CA 93117, USA). All measurements were taken in air and at room temperature using the AFM AC mode. Silicon AFM tips (AC160TS, Oxford Instruments, Tokyo, Japan) of 300 kHz resonance frequency and 26 N/m nominal spring constant were used. Morphometric (roughness value) characterization was accomplished using IgorPro 6.37 software. For AFM measurements, dry primed seeds were immobilized in a home-made sample holder. For each treatment, a number of 2D and 3D images were recorded (*n* = 17–22).

#### 3.2.3. Micro-Raman Spectroscopy on Seeds

Micro-Raman spectroscopy was conducted using a Renishaw In-Via equipped with a CCD camera electrically cooled laser line at 785 nm with a laser power between 3 and 30 mW, and a diffraction grating with 1200 lines/mm. In order to improve signal/noise ratio, spectra were acquired with 10 s of integration time and 10 accumulations.

### 3.3. Physiological Measurements

#### 3.3.1. Leaf Pigments

Physiological measurements were performed on intact 14-day-old plants. The total Chl and flavonoid content of intact leaves was determined by means of a Dualex instrument (ForceA, Orsay, France) on the abaxial leaf surface. The Chl/flavonoid ratio was used to estimate the nitrogen balance index (NBI).

#### 3.3.2. Photosynthetic Gas Exchange

Leaf gas exchange was evaluated by an LCpro+ photosynthesis system (ADC Bioscientific Ltd., Hoddesdon, Herts, UK). The measurements were performed on intact leaves enclosed into a leaf chamber at 800 μmol m^−2^ s^−1^ photosynthetic photon flux density at the leaf level, 200 μmol s^−1^ of air flow rate, 400 μmol mol^−1^ of CO_2_ concentration; leaf temperature and relative air humidity were set to 25 °C and 55–60%, respectively. Net photosynthetic rate (*A_N_*) and stomatal conductance (*g_s_*) were calculated using ADC software PRD-1054 ver. 1.09, and the intrinsic water use efficiency (*iWUE*) was calculated as the ratio between *A_N_* and *g_s_*.

#### 3.3.3. Chlorophyll Fluorescence

Chl fluorescence parameters were measured by a MAXI-Imaging-PAM chlorophyll fluorometer (Walz, Effeltrich, Germany). Minimal (*F*_0_) and maximal (*F_m_*) Chl fluorescence were determined in 20 min dark-adapted plants and used to calculate the maximum quantum yield of photosystem II (*F_v_/F_m_* = (*F_m_ − F*_0_*)/F_m_*). Then, leaves were exposed to an actinic light (180 μmol m^−2^ s^−1^), and saturating pulses (over 6000 μmol m^−2^ s^−1^) with a duration of 0.8 s were applied to determine steady-state (*F*) and maximum (*F_m_′*) fluorescence in the light. The quantum yield of photosystem II photochemistry was calculated according to [37] and the non-photochemical dissipation of absorbed light energy (*NPQ*) was determined according to [38].

### 3.4. Thylakoid Membrane Studies

#### 3.4.1. Preparation and Fractionation

Isolation of thylakoid membranes was performed as in [39], utilizing 30 min dark-adapted well-developed 2nd and 3rd leaf pairs from 14-day-old seedlings. The thylakoids were supplied with 30% (*v*/*v*) glycerol and stored at −20 °C until future use. Before each experiment, the membranes were washed in a measuring medium containing 20 mM Tricine, 250 mM sorbitol, 5 mM MgCl, pH 7.6. Chl concentration was determined spectrophotometrically (Specord 50 Plus spectrometer, Analytic Jena, Germany) according to [40]. Thylakoid fractionation was achieved as in [41] with slight modification. After two washing steps, the Chl concentration of the thylakoids containing pellets was adjusted to 0.1 mg Chl/mL, and the membranes were fractionated with 0.5% digitonin. After 15 min incubation and slow stirring, the samples were diluted 6.7 times and centrifuged for 30 min at 10,000× *g*. The supernatants were discarded, and the Chl concentration and total Chl content of the grana containing pellets were determined as a percentage from the total Chl concentration of the initial thylakoid sample.

#### 3.4.2. Lipid Peroxidation Evaluation

The lipid peroxidation of isolated thylakoids was assayed by detecting the levels of MDA as in [42]. The absorption at 532 nm was corrected for non-specific scattering at 600 nm, and the respective molar extinction coefficient of MDA (155 mM/cm) was used to estimate the MDA concentration.

### 3.5. Statistical Evaluation

Data are presented as mean and its standard error (SE). When applicable, the statistical significance was determined by a paired sample Wilcoxon signed ranks test, with *p* = 0.05 performed via Origin 2018 software.

## 4. Conclusions

The role of nanotechnology in agriculture is continuously increasing, and nanomaterials are considered environmentally friendly for enhancing global production and food quality. SWCNT, with their spectroscopic and electronic properties, have emerged as promising nanoparticles that could offer possibilities for precision agriculture. The present study reveals that the effect of seed priming with P85-SWCNT spans over different stages of plant development and that the observed decreased stomatal conductance and increased intrinsic water use efficiency of 14-day-old seedlings are preceded by an altered seed topology observed right after the priming procedure, followed by impaired seed germination on the fifth day of growth. Nevertheless, our data demonstrate that P85-SWCNT seed priming can be regarded as generally non-toxic for pea plants, since they largely preserve their optimal photosynthetic capability. It would be interesting to further explore how the observed mechanical damage to the seed coat induced by these nanomaterials during the priming procedure might be related to the uptake of water-insoluble soil components and if this can have some advantageous effects on the plant metabolism, especially under stress conditions. This could allow for an enhanced uptake of supplements, nutrients, and biostimulants through the seed coat, which might be beneficial for early plant growth and development—a hypothesis that requires verification.

## Figures and Tables

**Table 1:** Germination parameters determined for the studied treatments for a period of 5 days: germinability (*G*), mean germination time (*MT*), synchrony of germination (*Z*), and mean rate of germination (*MR*). Means ± SE. Differences from hydro-primed samples were assessed by a paired sample Wilcoxon signed ranks test and are indicated by an asterisk (*) when significantly different at *p* < 0.05.

Priming Treatment	*G* (%)	*MT* (Days)	*Z*	*MR* (Germinated Seeds/Day)
H_2_O	75.4 ± 5.5	2.47 ± 0.15	0.35 ± 0.05	0.42 ± 0.03
10 mg/L P85-SWCNT	68.1 ± 4.3 *	3.20 ± 0.12 *	0.40 ± 0.05 *	0.32 ± 0.01 *
50 mg/L P85-SWCNT	61.2 ± 3.5 *	3.31 ± 0.07 *	0.36 ± 0.06 *	0.30 ± 0.01 *

**Table 2:** Leaf total chlorophyll (Chl) and flavonoid abundance, and nitrogen balance index (NBI) in pea plants developed from hydro- and P85-SWCNT-primed seeds. Measurements were performed on intact 14-day-old seedlings. Means ± SE (*n* = 30). Differences from hydro-primed samples were assessed by a paired sample Wilcoxon signed ranks test and are indicated by an asterisk (*) when significantly different at *p* < 0.05.

Priming Treatment	Chl	Flavonoid	NBI
H_2_O	28.4 ± 0.5	0.511 ± 0.041	55.8 ± 0.9
10 mg/L P85-SWCNT	28.0 ± 0.5	0.522 ± 0.052	54.0 ± 1.0 *
50 mg/L P85-SWCNT	27.3 ± 0.4	0.515 ± 0.005	53.3 ± 0.9 *

**Table 3:** Net photosynthetic rate (*A_N_*), stomatal conductance (*g_s_*), and intrinsic water use efficiency (*iWUE*) of pea plants developed from hydro- and P85-SWCNT-primed seeds. Measurements were performed on intact 14-day-old seedlings. Means ± SE (*n* = 9). Differences from hydro-primed samples were assessed by a paired sample Wilcoxon signed ranks test and are indicated by an asterisk (*) when significantly different at *p* < 0.05.

Priming Treatment	*A_N_* (μmol m^−2^ s^−1^)	*g_s_* (mol m^−2^ s^−1^)	*iWUE* (μmol mol^−1^)
H_2_O	15.3 ± 0.3	0.32 ± 0.02	48.7 ± 3.0
10 mg/L P85-SWCNT	14.5 ± 0.3 *	0.26 ± 0.01 *	57.4 ± 2.8 *
50 mg/L P85-SWCNT	14.2 ± 0.3 *	0.27 ± 0.02 *	56.7 ± 4.9 *

**Table 4:** PAM fluorescence parameters determined on intact leaves of pea plants developed from hydro- and P85-SWCNT-primed seeds: maximum quantum yield of photosystem II in dark-adapted state (*F_v_/F_m_*), quantum efficiency of photosystem II photochemistry (*Φ_PSII_*), non-photochemical quenching (*NPQ*), fraction of open photosystem II reaction centers (*q_L_*). Measurements were performed on intact 14-day-old seedlings. Means ± SE (*n* = 9). Differences from hydro-primed samples were assessed by a paired sample Wilcoxon signed ranks test and are indicated by an asterisk (*) when significantly different at *p* < 0.05.

	Priming	H_2_O	10 mg/L P85-SWCNT	50 mg/L P85-SWCNT
Parameter				
*F_v_/F_m_*		0.794 ± 0.002	0.785 ± 0.003 *	0.791 ± 0.002
*Φ_PSII_*		0.520 ± 0.004	0.528 ± 0.003	0.526 ± 0.003
*NPQ*		1.089 ± 0.016	1.047 ± 0.017	1.132 ± 0.013
*q_L_*		0.589 ± 0.007	0.595 ± 0.006	0.614 ± 0.006 *

**Table 5:** Structural parameters and malondialdehyde (MDA) level (mean ± SE) determined for thylakoid membranes isolated from 14-day-old pea seedlings developed from hydro-, 10 mg/L and 50 mg/L P85-SWCNT-primed seeds (*n* = 4): Chl *a/b* ratio estimated for intact thylakoid and fractionated grana membranes, and Chl content of grana stacks relative to the total Chl content of the respective thylakoids.

Priming Treatment	Chl *a*/*b* in Thylakoids	Chl *a*/*b* in Grana	Chl in Grana (%)	MDA (µmol)
H_2_O	2.85 ± 0.02	1.97 ± 0.12	36.5 ± 6.7	0.343 ± 0.020
10 mg/L P85-SWCNT	2.85 ± 0.02	1.95 ± 0.12	35.4 ± 6.6	0.319 ± 0.017
50 mg/L P85-SWCNT	2.86 ± 0.02	1.90 ± 0.10	31.7 ± 7.0	0.400 ± 0.012

## Data Availability

All data are contained within the manuscript and are available upon request.
